# Antiviral Activities of High Energy E-Beam Induced Copper Nanoparticles against H1N1 Influenza Virus

**DOI:** 10.3390/nano12020268

**Published:** 2022-01-14

**Authors:** Taesung Ha, Thi Tuyet Mai Pham, Mikyung Kim, Yeon-Hee Kim, Ji-Hyun Park, Ji Hae Seo, Kyung-Min Kim, Eunyoung Ha

**Affiliations:** 1 Department of Nano Chemical Materials Engineering, Korea National University of Transportation, Chungju 27469, Korea; river@srsrad.com; 2 Seoul Radiology Services Co., Seoul 02050, Korea; kimyhc@srsrad.com (Y.-H.K.); jhpark@srsrad.com (J.-H.P.); 3 Department of Biochemistry, School of Medicine, Keimyung University, Deagu 42601, Korea; phamtmai6@gmail.com (T.T.M.P.); balee96@naver.com (M.K.); seojh@dsmc.or.kr (J.H.S.)

**Keywords:** copper, nanoparticle, influenza virus, antiviral activity, electron beam

## Abstract

The pandemic outbreak of COVID-19 in the year of 2020 that drastically changed everyone’s life has raised the urgent and intense need for the development of more efficacious antiviral material. This study was designed to develop copper nanoparticles (Cu NPs) as an antiviral agent and to validate the antiviral activities of developed copper NP. The Cu NPs were synthesized using a high energy electron beam, and the characteristic morphologies and antiviral activities of Cu NPs were evaluated. We found that Cu NPs are of spherical shape and uniformly distributed, with a diameter of around 100 nm, as opposed to the irregular shape of commercially available copper microparticles (Cu MPs). An X-ray diffraction analysis showed the presence of Cu and no copper oxide II and I in the Cu NPs. A virus inactivation assay revealed no visible viral DNA after 10- and 30-min treatment of H1N1 virus with the Cu NPs. The infectivity of the Cu NPs-treated H1N1 virus significantly decreased compared with that of the Cu MPs-treated H1N1 virus. The viability of A549 bronchial and Madin-Darby Canine Kidney (MDCK) cells infected with Cu NPs-treated H1N1 was significantly higher than those infected with Cu MPs-treated H1N1 virus. We also found cells infected with Cu NPs-treated H1N1 virus exhibited a markedly decreased presence of virus nucleoprotein (NuP), an influenza virus-specific structural protein, compared with cells infected with Cu MPs-treated H1N1 virus. Taken together, our study shows that Cu NPs are a more effective and efficacious antiviral agent compared with Cu MPs and offer promising opportunities for the prevention of devastatingly infectious diseases.

## 1. Introduction

In the year 2020, the world experienced the pandemic outbreak of COVID-19, a contagious disease caused by severe acute respiratory syndrome-coronavirus 2 (SARS-CoV-2) [1]. Contrary to everyone’s expectation that the outbreak would subside soon, COVID-19 continued to the year 2021. Extraordinary public health measures have been implemented worldwide to prevent the virus from spreading, ranging from transport restriction and quarantine to social distancing and isolation [2]. This unprecedented calamity has raised the urgent and intense need for the development of antiviral materials with the highest efficacy to keep highly contagious viruses at bay [3]. One such candidate is copper (Cu) because, compared to other antimicrobial and antiviral materials, such as silver and gold, copper is cheaper and more easily accessible, which makes it very favorable as a commodity for daily application [4].

Cu is an essential trace element. It transmits electricity and transfers heat with the highest conductivity amongst any metal [5]. Cu is an essential micronutrient. It catalyzes heme synthesis and iron absorption that sustains lives [6]. Cu is also an antimicrobial agent. Its usage as an antimicrobial agent by human civilization dates back between 2600 and 2200 B.C. [7,8]. The Egyptians used Cu to sterilize chest wounds and to purify drinking water [8]. The Romans, Greeks, and others followed and used Cu for the treatment of infections and for hygienic purposes [8].

The use of Cu in medicine continued, and, today, the antimicrobial activities of Cu are firmly established [7,9,10,11]. The Environmental Protection Agency officially announced Cu and its alloys as antimicrobial metallic agents in 2008 (http://archive.epa.gov/pesticides/registration/web/pdf/copper_red_fs.pdf, access on 2 November 2021). Cu and Cu alloys show a broad spectrum of antimicrobial activities against various bacteria, such as E. coli, S. aureus, P. aeruginosa, and K. aerogenes [12,13,14]. The antiviral activities of Cu, however, have not been well established. Moreover, the antiviral activities of Cu have not been elucidated as much as those of other antiviral agents, such as silver.

Like Cu, silver has been used to prevent infections since antiquity [15]. The antiviral effect of silver was evaluated and confirmed in Newcastle disease virus-infected chicken eggs [16]. Moreover, the antiviral activities of silver against influenza virus A, herpes simplex viruses, and HIV-1 virus were extensively demonstrated [17,18,19,20,21,22]. Curcumin extract-derived silver showed highly efficient antiviral activity against respiratory syncytial virus [23]. In addition, studies showed silver-induced apoptosis reflected by caspase 3 activation, DNA fragmentation, and chromatin condensation [17,24].

We previously showed enhanced conductivity of high energy electronic beam (E-beam)-induced Cu nanoparticles (Cu NPs) [25]. The synthesis of E-beam-induced Cu NPs, compared to commercially available Cu microparticles (Cu MPs) synthesized by mechanical milling, requires fewer procedural steps and less time, which makes them suitable for mass production. The size of E-beam-induced Cu NPs can also be adjusted by varying the irradiation dose or dose rate. We reasoned, based on the enhanced conductivity of E-beam-induced Cu NPs, that E-beam-induced Cu NPs would show effective and efficacious antiviral activities. Thus, with the urgent necessity to establish the antiviral role of Cu, we designed and conducted the current study.

## 2. Materials and Methods

### 2.1. Materials

All aqueous solutions were prepared using deionized water (Milipore, Merck, Seoul, Korea, specific resistivity ≥18.2 ΜΩ cm) and ethylene glycol (EG, JUNSEI, Tokyo, Japan) with a ratio of 50:50. Cu sulphate pentahydrate (CuSO_4_:5H_2_O, 99%, Sigma Aldrich, Seoul, Korea) was used as a metal salt, and polyvinylpyrrolidone (PVP) (Mw = 360,000) was used as a dispersing agent. The isopropyl alcohol (IPA, 99% DUKSAN, Seoul, Korea) was used as a radical scavenger to remove hydroxyl radical. Cu MPs that had been synthesized primarily by mechanical jet milling were purchased from K H Tech, Incheon, Korea.

### 2.2. Synthesis of Cu NPs

**Figure 1:**
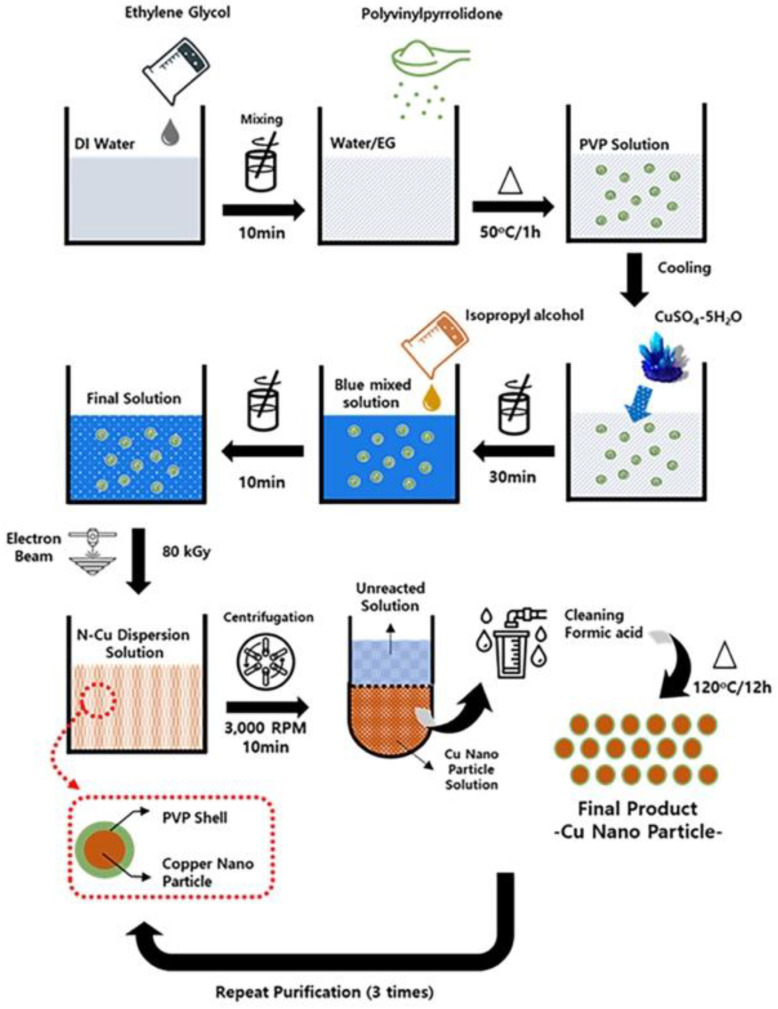
Preparation procedures of copper nanoparticles (Cu NPs).

Radiolytic method was used for the synthesis of Cu NPs using 10 MeV of high energy E-beam. Deionized water and ethylene glycol (50:50 ratio) were mixed in a 100 mL beaker. CuSO_4_:5H_2_O (0.05 M) was dispersed in the solution, followed by the addition of 1.2 g of dispersing agent and 2 M of isopropyl alcohol. After the mixture was stirred with the mechanical stirrer at 500 rpm for 1 h, oxygen in the solution was removed by N_2_ gas. The reaction mixture was transferred to a culture, then irradiated using E- beam (10 MeV, 20 kW). The absorbed dose was 80 kGy and dose rate was 2 kGy/s. The obtained Cu NPs suspension was centrifuged at 7000 rpm for 20 min and the clear supernatant solution was discarded. The sediments were washed by formic acid several times then desiccated at 40 °C under vacuum desiccator. The overall process of the synthesis of Cu NPs is illustrated in Figure 1.

### 2.3. Characterization of Cu NPs

The phase analysis of the Cu NPs was conducted by X-ray powder diffraction (XRD, Smartlab, Rigaku Corporation, Tokyo, Japan) with Cu Kα radiation (λ = 1.54178 Å). The morphologies of the particles were investigated using the field-emission scanning electron microscope (SEM, Sirion, FEI, Hillsboro, OR, USA) with accelerating voltage of 30 kV equipped with an energy dispersive spectroscopy (EDS) and the field emission transmission electron microscopy (TEM, JEM-2100F HR, JEOL Ltd., Tokyo, Japan) with accelerating voltage of 200 kV.

### 2.4. Cell Culture and Virus Propagation

A549 adenocarcinomic human alveolar basal epithelial and Madin-Darby Canine Kidney (MDCK) cells were purchased from Korean Cell Line Bank and were maintained in monolayer in Roswell Park Memorial Institute Medium (RPMI) and Dulbecco’s Modified Eagle Medium (DMEM), respectively, supplemented with 10% fetal bovine serum (FBS) at 37 °C and 5% CO_2_.

Influenza A (H1N1) virus was purchased from Korean Bank for Pathogenic Viruses. Viruses were propagated in MDCK cells. One day before inoculation, MDCK cells were seeded in T75 flask. When the cells were at 80–90% confluence, cells were infected with virus and incubated for 2 h at 37 °C. After infection, cells were washed with PBS, followed by addition of virus growth media containing MEM, BSA 7.5% (Sigma-Aldrich, St. Louis, MO, USA), TPCL-trypsin 2 μg/mL (Sigma-Aldrich, St. Louis, MO, USA). When 80–90% of cells detached from the flask, culture medium was collected and centrifuged at 1500 rpm for 5 min. Supernatant containing viruses was collected and stored at −80 °C.

### 2.5. Fifty Percent Tissue Culture Infectious Dose (TCID_50_) Assay

TCID_50_ was used to measure the titration and infectivity of viruses. Briefly, cells were seeded into 96-well plates and incubated for 24 h. Viruses were incubated with the absence or presence of Cu MPs and Cu NPs for 10 min, and 100 µL of incubated samples was used to infect cells as described in Section 2.6. After infection, cells were incubated for additional 48 h. After 48 h, cells were washed with phosphate buffered solution (PBS), fixed with 4% paraformaldehyde-PBS (Fujifilm, Osaka, Japan) for 20 min, and stained with crystal violet solution for 10 min at room temperature (RT). The TCID_50_ per mL was calculated according to Reed and Muench method [26].

### 2.6. Virus Infection

MDCK or A549 adenocarcinomic human alveolar basal epithelial cells were seeded into 6-well culture plates for 20–24 h at 37 °C and 5% CO_2_ before infection. When the confluence of cells reached 80–90%, 200 μL of H1N1 (TCID_50_/mL = 1.2 × 10^10^) in PBS containing 0.1mM CaCl_2_, 0.1 mM MgCl_2_, 100 U/mL penicillin 0.1 mg/mL streptomycin, 7.5% BSA (Sigma-Aldrich, St. Louis, MO, USA), TPCL-trypsin 2 μg/mL (Sigma-Aldrich, St. Louis, MO, USA) were added into the cells and incubated for 1 h. After infection, viral solutions were removed, cells were washed briefly by PBS, and cell culture media were added into cells.

### 2.7. Virus Inactivation Assay

Cu MPs and Cu NPs were dispersed in PBS (5% *w*/*v*). To evaluate the antiviral activities, Cu MPs and Cu NPs solutions were added to virus solution (TCID_50_/_mL_ = 1.2 × 10^10^) to the final concentration of 0.5% *w*/*v* and incubated for 0, 10, and 30 min. After incubation of Cu MPs or Cu NPs with virus for the indicated time, viral RNA was isolated using RibospinTM vRD (GeneAll, Seoul, Korea) as recommended by the manufacturer.

### 2.8. Cellular RNA Isolation and Polymerase Chain Reaction (PCR) Analysis

Total RNA in cells was isolated using TRIzol Reagent (Life Technology, Carlsbad, CA, USA), as recommended by the manufacturer. RNA purity and concentration were determined by ASP2680 spectrophotometer (ATCGene, Madison, CT, USA). PCR analysis was conducted using 5X Green Gotaq Flexi Buffer (Promega, Madison, WI, USA) and 1.2% of agarose gel electrophoresis was used to separate DNA fragments. Primers for H1N1 influenza virus-specific hemagglutinin (HA) protein were used to determine H1N1 virus. Expression levels of cellular H1N1 virus HA protein were normalized against β-actin, an internal control of A549 and MDCK cells. Primers for β -actin, human and canine, and HA protein are as follows:

HA protein (H1N1 virus) Forward 5′-CCCAGGRTATTTCKCCGAYTATGAGG-3′

Reverse 5′-TACCATTCCAGTCCACCCCCTTCA-3′

β-actin (human) Forward 5′-GGACTTCGAGCAAGAGATGG-3′

Reverse 5′-AGCACTGTGTTGGCGTACAG-3′

β-actin (canine) Forward 5′-GCGCAAGTACTCTGTGTGGA-3′

Reverse 5′-AAAGCCATGCCAATCTCATC-3′

### 2.9. Cell Viability Assay

Cells (2 × 10^4^ cells/mL) were seeded into 96-well plates and were incubated at 37 °C and 5% CO_2_ for 24 h. Viruses were incubated with Cu MPs or Cu NPs suspensions as described in Section 2.7 for indicated times and then added to cells for infection. Twenty-four hours after infection, 10 µL Cell Counting Kit-8 (Dojindo, Rockville, MD, USA) reagent was added to each well and the cells were incubated for 1 h at 37 °C. The optical density was measured at 450 nm by a microplate spectrophotometer. Morphological characterization was analyzed using inverted microscope Olympus CKX53 (Olympus, Tokyo, Japan) with different magnifications and digitally visualized using software iSolution Auto plus (IMT Inc., Daejeon, Korea). For the evaluation of Cu MPs and Cu NPs cytotoxicity, Cu MPs and Cu NPs were first dispersed in PBS (5% *w*/*v*), added to cells to the final concentration of 0.5% *w*/*v*, and incubated for the indicated time.

### 2.10. Western Blot and Coomassie Blue Staining

Cu MPs and Cu NPs solutions were added to virus solution to the final concentration of 0.5% *w*/*v* and incubated for 0, 10, and 30 min. After the incubation, samples were ultracentrifuged at 100,000× *g* for 2 h at 4 °C. Obtained samples were lysed in RIPA buffer (Thermo Fisher Scientific, Rockford, IL, USA). Five micrograms of protein from each sample were resolved by sodium dodecyl sulphate-polyacrylamide gel electrophoresis (SDS-PAGE) gels and then stained with Coomassie blue or analyzed by immunoblotting. Anti-nucleoprotein (NuP) (Abcam, Cambridge, UK) was used to detect the presence of virus.

### 2.11. Confocal Microscopy

Confocal microscopy was used to observe the localization of influenza H1N1 virus and to visually determine the antiviral effects of Cu MPs and Cu NPs in MDCK or A549 cells. After being incubated with Cu MPs or Cu NPs for the indicated times, viruses were used to infect cells for 1 h. Then, cells were washed with PBS and fixed in paraformaldehyde PBS (Fujifilm, Osaka, Japan) for 20 min at RT. Cells were then permeabilized with 0.1% Triton X-100 in PBS for 10 min and blocked with 1% bovine serum albumin (BSA) in PBS-T for 30 min at RT. After blocking, cells were incubated with mouse monoclonal antibody against viral NuP (1:200) (Abcam, Cambridge, UK) overnight at 4 °C and goat antibody mouse (1:1000) (Thermo Fisher Scientific, Rockford, IL, USA) for 1 h at RT. Nuclei were stained using 4′,6-diamidino-2-phenylindole (DAPI). Fluorescence was visualized with a Carl Zeiss LSM5 EXCITER fluorescence microscope (Carl Zeiss, Oberkochen, Germany).

## 3. Results and Discussions

### 3.1. Morphological Analysis of Cu NPs and Cu MPs

**Figure 2:**
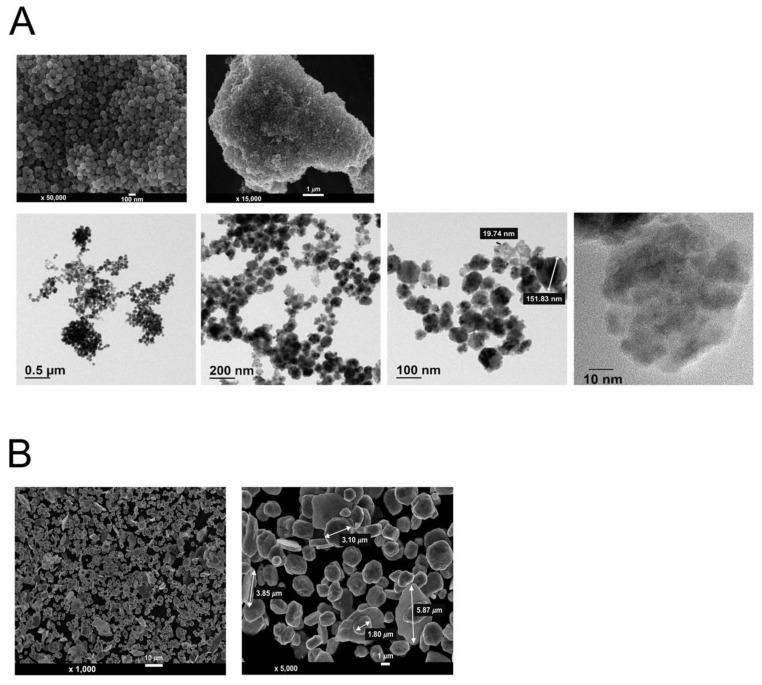
Scanning electron microscopy (SEM) and transmission electron microscopy (TEM) images of copper (Cu) nanoparticles (Cu NPs) synthesized by high energy E-Beam irradiation (**A**) and SEM images of Cu microparticles (Cu MPs) (**B**).

Previous studies suggested that the dose for the synthesis of Cu NPs with a uniform and nano-dimensional shape requires over 300 kGy [25,27,28]. However, we found in the current study that 80 kGy is required when a high energy E-beam (10 MeV) is used to produce a high-yield and consistent, as well as reproducible, synthesis of Cu NPs. Over various methods for the synthesis of Cu NPs, including vacuum vapor deposition, microemulsion, mechanical milling, electrolysis, and aqueous solution reduction [29,30,31,32,33], the E-beam-induced synthesis of Cu NPs is advantageous in that the shape and size can be controlled, as reflected in Figure 2A.

We employed SEM to confirm and validate the size and morphology of Cu NPs synthesized by 10 MeV high energy E-beam. The upper panels of Figure 2A show SEM images of Cu NPs synthesized by high energy E-beam. The shape of the particles is spherical, and the sizes of the particles are even with the diameter of about 100 nm as compared to the larger sizes in the dimension of μm and the irregular shape of Cu MPs (Figure 2B). The size and shape of Cu MPs are in line with a previous study [34].

We also performed further morphological analysis using TEM. The lower panels of Figure 2A show that the size and shape of Cu NPs are consistent with those of SEM analysis. The magnified TEM image with the scale of 10 nm revealed that the Cu NPs with the size of 100 nm are agglomerated with smaller nanoparticles of 10 to 20 nm in size.

As shown in Figure 2, most Cu NPs and Cu MPs are spherical. Thus, the surface-to-volume ratio of the Cu NPs and Cu MPs is 3/r, where r is the radius [35]. As r decreases, the surface-to-volume ratio increases. Assuming the radius of Cu MPs is 3.0 μm and that of agglomerated Cu NPs is 100 nm, the reactive surface of Cu NPs is 30 times higher than that of Cu MPs for the same volume. Thus, with the radius of individual Cu NPs being approximately 10–20 nm, the surface-to-volume ratio of Cu NPs would increase up to 150 to 300 times that of Cu MPs.

### 3.2. Characteristic Analysis of Cu NPs

Due to their superb electrical conductivity, low cost, and excellent biocompatibility, Cu and Cu-based compounds have been gaining much attention in the areas ranging from future nanodevices to control of infectious diseases in healthcare [36,37]. Despite the above-mentioned advantages, however, the synthesis of Cu NPs is very challenging since Cu NPs are easily oxidized when exposed to the air and unstable in solution. The additional advantage of the E-beam-induced synthesis of Cu NPs is that irradiation can generate appropriate reducing radicals without the production of any byproducts [28].

**Figure 3:**
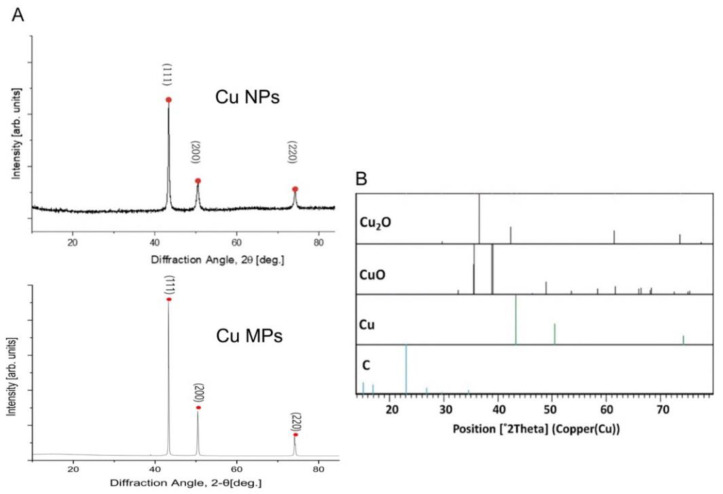
X-ray powder diffraction (XRD) pattern of copper (Cu) nanoparticles (Cu NPs) and microparticles (Cu MPs) (**A**) and standard card of Joint Committee on Powder Diffraction Standards (JCPDS) (**B**). Pure Cu file No. 04-0836, CuO_2_ file No. 05-0667, CuO file No. 48-1548.

**Figure 4:**
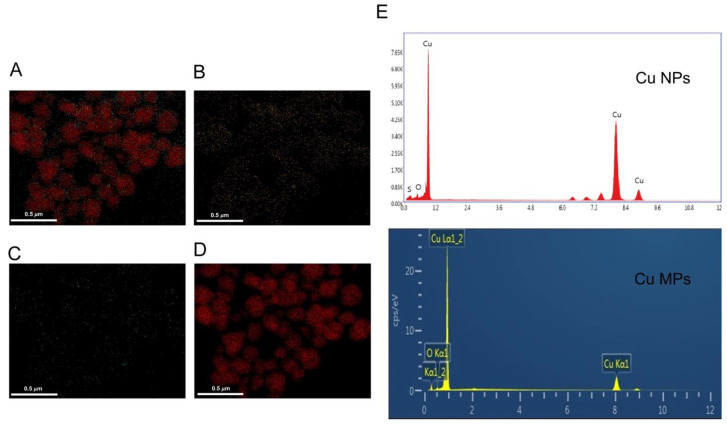
Energy dispersive spectroscopy (EDS) elemental spectrum and quantification results of copper (Cu) nanoparticles (Cu NPs). The EDS mapping of Cu NPs shows overlay (**A**), oxygen (**B**), sulfur (**C**), and Cu (**D**) distributions in micrographs, respectively. The EDS spectrum reveals the main components (oxygen, sulfur, and Cu) of Cu NPs and Cu microparticles (Cu MPs) (**E**).

XRD is a powerful technique to identify the crystalline phase present in the materials and to determine the structural properties, such as strain state, grain size, phase composition, preferred orientation, and structural defect, of the phase. Cu is highly susceptible to oxidation and easily converted into Cu impurities, such as Cu oxide II (CuO) and Cu oxide I (Cu_2_O). Thus, we investigated if Cu NPs and Cu MPs contain CuO or Cu_2_O by XRD analysis. The diffraction angles (2θ) of the XRD analysis of Cu NPs and Cu MPs in Figure 3A correspond to those of pure Cu in Figure 3B (peak values 2θ = 43.3, 50.3, 74.2 degrees in Figure 3A), respectively, indicating Cu NPs and Cu MPs contain no Cu oxide impurities (CuO or Cu_2_O). Further investigation of Cu NPs and Cu MPs by EDS analysis, an analytical technique used for the elemental analysis, also reveals the main components of Cu NPs and Cu MPs are Cu (~82%, Cu), suggesting that Cu ions in Cu NPs and Cu MPs are in the state of reduced form, not in the state of oxide form (Figure 4).

### 3.3. Virus Inactivation by Cu NPs

The antiviral activities of Cu have not been well exploited. Research regarding the antiviral role of Cu is still in its early stage and under intensive investigation. Recent studies showed the antiviral effects of Cu oxide against hepatitis C and herpes simplex viruses [38,39]. Other studies reported antiviral activities of Cu compounds against influenza viruses [40,41,42]. One possible antiviral mechanism of Cu is to inhibit viruses to enter and bind to host cells via interacting with the membrane glycoproteins of host cells. Another potential antiviral mechanism of Cu is to inactivate and degrade viral particles primarily via the generation of reactive oxygen species (ROS), leading to the degradation of the viral genome and proteins [43,44].

**Figure 5:**
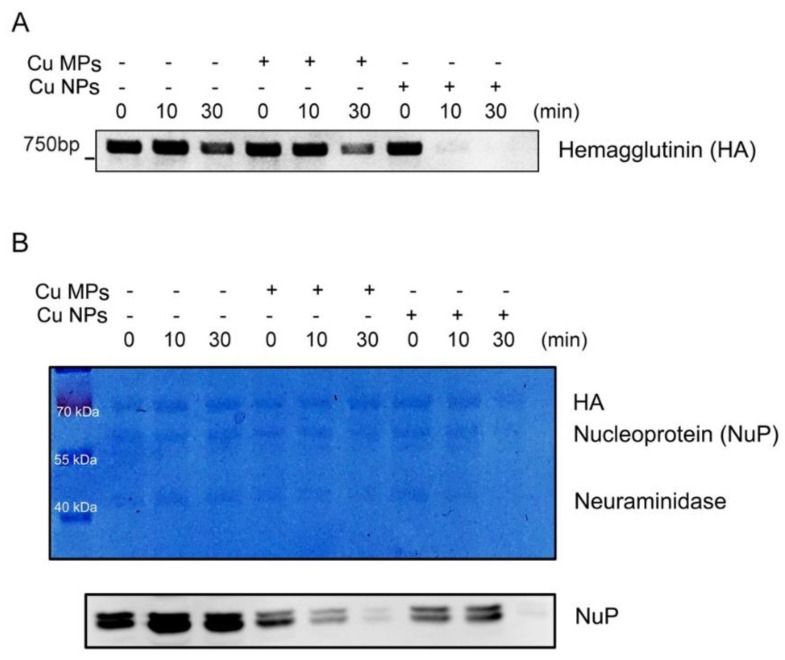
The antiviral effects of copper (Cu) microparticles (Cu MPs) and Cu nanoparticles (Cu NPs) against H1N1 virus. (**A**) Viral inactivation assay. H1N1 virus was incubated with the absence or presence of Cu MPs or Cu NPs between 10 min and 30 min and analyzed for virus-specific heme agglutinin (HA) gene by PCR analysis. (**B**) Coomassie bule staining (upper panel) of viral proteins and western blot analysis (lower panel) of viral nucleoprotein (NuP) from viral inactivation assay (1, 10, and 30 min).

Herein, with Cu NPs that we developed as more efficient and efficacious antiviral material, we then determined the efficacy of the antiviral activities of Cu NPs and possible antiviral mechanism of Cu NPs. We used commercially available Cu MPs as a positive control. Figure 5A exhibits the viral inactivation activities of Cu MPs and Cu NPs and degradation of the viral genome, HA gene. The expressions of the viral HA gene in the control, 0 min, as expected, decreased in proportion to the incubation time since the viability of the virus is proportional to the time of exposure to open-air environment. However, the Cu MPs treatment into the H1N1 suspension decreased the expressions of the viral HA gene more profoundly at 30 min than the control, suggesting that the virus is inactivated and viral particles degraded by Cu MPs. To our expectation that the larger surface area of Cu NPs would exert stronger anti-viral activity, the expressions of the viral HA gene in Cu NPs at 10 and 30 min were not visible, implicating that the virus is strongly inactivated by Cu NPs. The expressions of the viral HA gene in the Cu NPs-treated virus markedly decreased at 10 and 30 min compared with those in the Cu MPs-treated virus. These data clearly demonstrate that the antiviral activity of Cu NPs against H1N1 influenza virus is superior to that of Cu MPs.

To validate further that the virus is indeed inactivated and degraded, we then determined the viral proteins by western blot analysis (Figure 5B). The upper panel of Figure 5B represents Coomassie blue-stained viral proteins, HA, NuP, and neuraminidase [45]. The lower panel of Figure 5B represents the western blot analysis of NuP. Almost no protein bands are visible at 30 min of Cu NPs in both the Coomassie blue and western blot analyses, indicating the degradation of viral particles.

### 3.4. Infectivity of Cu NPs-Treated Virus

**Figure 6:**
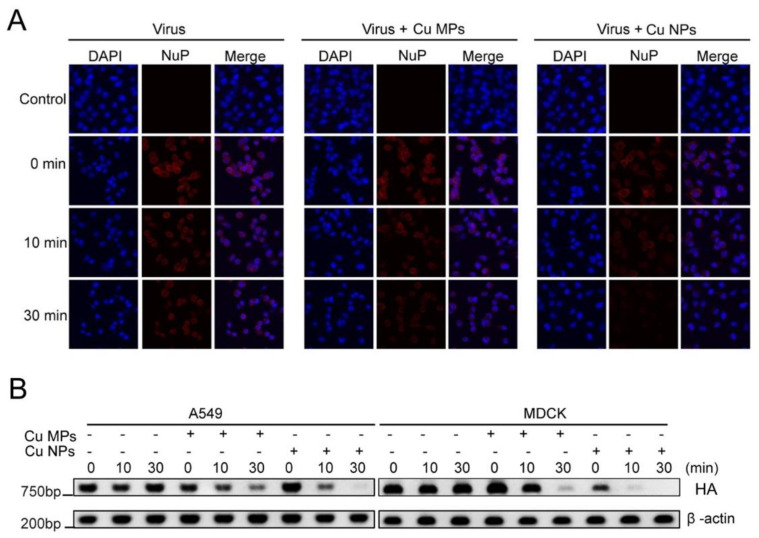
Copper (Cu) microparticles (Cu MPs) and Cu nanoparticles (Cu NPs) induce decrease in NuP protein expression in infected cells. (**A**) Immunofluorescence confocal microscopy assay for NuP protein expression (red) in virus infected MDCK or A549 cells with the absence or presence of Cu MPs or Cu NPs at 24 h post infection. The cell nuclei were stained with DAPI (blue). (**B**) Standard PCR assay for viral hemagglutinin (HA) gene. A549 and MDCK cells were treated with samples from viral inactivation assay (1, 10, and 30 min) and incubated for 24 h. Cellular RNA were extracted and analyzed by PCR assay for virus-specific HA gene.

To further evaluate the anti-viral activity of Cu NPs compared to that of Cu MPs, we next determined the presence of cellular viral particles by confocal microscopy. MDCK cells were infected with H1N1 virus, Cu MPs-treated virus, and Cu NPs-treated virus (Figure 6A). We used virus-specific NuP antibody to detect the presence of influenza virus. NuP is a highly conserved virus-specific protein that is most abundantly present in infected cells [46]. In the uppermost panels of Figure 6A, NuP protein is not visible in control cells that are not infected with virus, as expected. In the second upper panels of Figure 6A, 0 min, cells infected with virus only showed a strong presence of NuP, and the treatment of the virus with Cu MPs and Cu NPs attenuated the presence of NuP around the cellular nucleus. Moreover, we observed less intensity of the fluorescence of NuP in cells infected with Cu NPs-treated virus than those infected with Cu MPs-treated virus. Of note, the intensities of fluorescence of NuP at 30 min of cells infected with Cu NPs-treated virus are similar to those of cells without virus infection, evidence that suggests a very effective antiviral activity of Cu NPs. 

We then also evaluated the expressions of viral genome HA gene (Figure 6B) and observed consistent results with those of Figure 6A. The expressions of viral DNA in both A549 and MDCK cells infected with Cu NP-treated virus markedly decreased at 10 and 30 min compared with those infected with Cu MP treated virus. 

**Figure 7:**
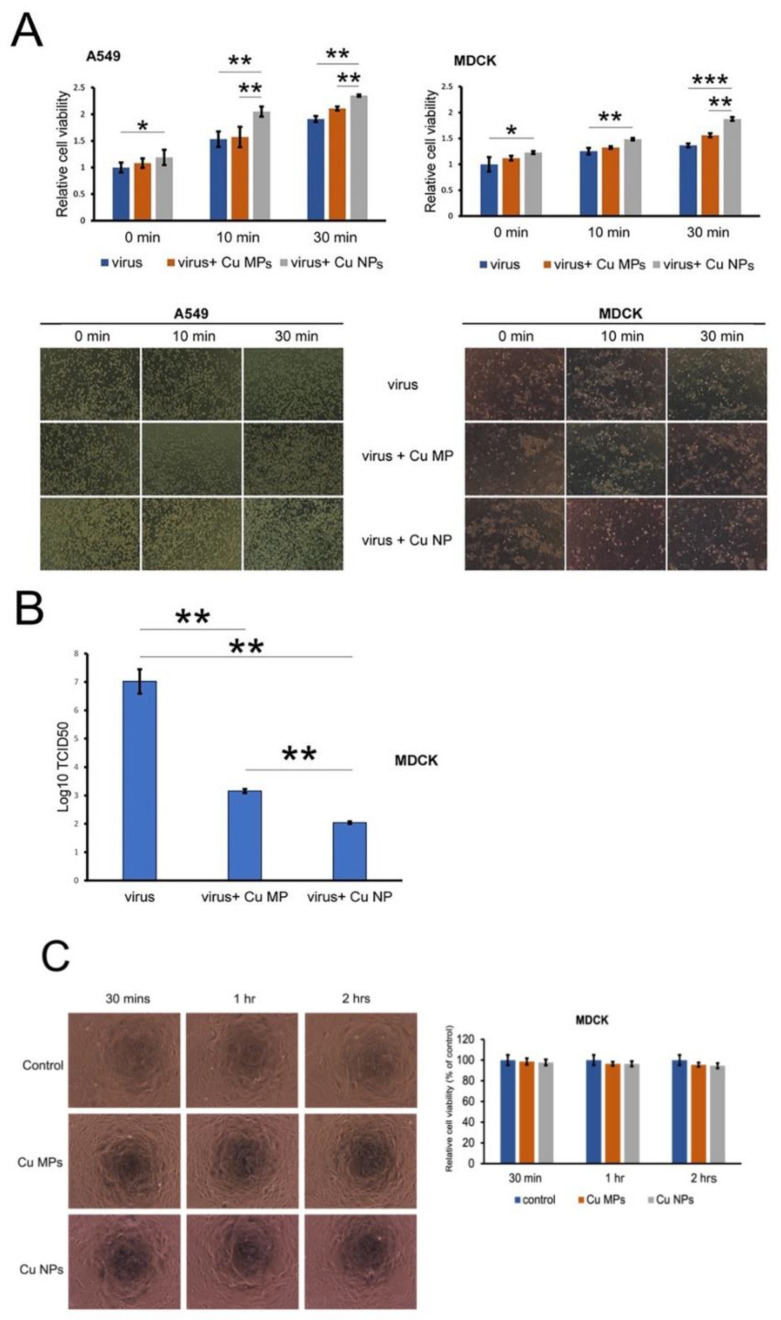
Copper (Cu) microparticles (Cu MPs) and Cu nanoparticles (Cu NPs) attenuate the cytopathic effect of H1N1 virus in A549 and MDCK cells. (**A**) Upper panels: cell viability assay for indicated cells. Lower panels: cell morphologies for indicated cells (magnification: 100×). (**B**) TCID_50_ assay for MDCK cells. (**C**) Cytotoxicity of Cu MPs and Cu NPs. ***, *p* < 0.001, **, *p* < 0.01, *, *p* < 0.05.

Next, we determined the viabilities of A549 and MDCK cells infected with H1N1 virus, Cu MPs-treated virus, and Cu NPs-treated virus (Figure 7A). In line with the results from the viral inactivation assay, we found that the cell viabilities are significantly higher in both A549 and MDCK cells infected with Cu MPs- and Cu NPs-treated virus compared with those infected with virus alone. We also found that the cell viabilities are markedly higher in both A549 and MDCK cells infected with Cu NPs-treated virus compared with those infected with Cu MPs treated virus, another piece of evidence that the antiviral activity of Cu NPs is greater than that of Cu MPs. The lower panels of Figure 7A show cell morphologies consistent with the results from the upper panels of Figure 7A, cell viability assay. The TCID_50_ assay is an assay that is commonly used to quantify the number of infectious virus particles, the titration of the virus. We then determined the TCID_50_ values of the H1N1 virus alone, Cu MPs-treated H1N1 virus, and Cu NPs-treated H1N1 virus (Figure 7B) and found that the TCID_50_ value of Cu NPs-treated H1N1 virus is significantly lower than those of the virus alone and Cu MPs-treated H1N1 virus, indicating lower viral infectivity when treated with Cu NPs. Finally, we investigated the cytotoxicity of Cu MPs and Cu NPs (Figure 7C). Additionally, we found no significant differences in cytotoxicity among the control, Cu MPs-treated, and Cu NPs-treated samples. These results are expected given the size difference between the virus and eukaryotic cells. The typical size of H1N1 influenza virus is 80 to 120 nm in diameter [47] and that of eukaryotic cells in the dimension of μm, resulting in more than a million-fold difference in volume [48].

## 4. Conclusions

In the current study, we showed that 80 kGy, not over 300 kGy, is required to produce a high-yield and reproducible synthesis of Cu NPs when the high energy E-beam (10 MeV) is used. We also evaluated the antiviral activities of Cu NPs as compared to those of commercially available Cu MPs and demonstrated that Cu NPs show significantly higher, more efficient, and efficacious antiviral activities than Cu MPs, possibly due to a greater surface to volume ratio. The Cu NPs showed no visually present viral particles 30 minutes after contact with H1N1 virus, as evidenced by virus inactivation assay, western blot analysis, and confocal analysis. With the pandemic outbreak of COVID-19 in the year of 2020, we cannot exclude the possibility of another viral pandemic outbreak. Thus, the utilization of Cu NPs in the healthcare setting as well as in our everyday lives would greatly attenuate the risk of viral transmission, thereby contributing to the prevention of a potential pandemic outbreak of another virus in the future.

## Data Availability

The data presented in this study are available on request from the corresponding author.
